# Methodology of the DCCSS later fatigue study: a model to investigate chronic fatigue in long-term survivors of childhood cancer

**DOI:** 10.1186/s12874-021-01298-7

**Published:** 2021-05-16

**Authors:** Adriaan Penson, Sylvia van Deuren, Ewald Bronkhorst, Ellen Keizer, Tom Heskes, Marieke J. H. Coenen, Judith G. M. Rosmalen, Wim J. E. Tissing, Helena J. H. van der Pal, Andrica C. H. de Vries, Marry M. van den Heuvel-Eibrink, Sebastian Neggers, Birgitta A. B. Versluys, Marloes Louwerens, Margriet van der Heiden-van der Loo, Saskia M. F. Pluijm, Martha Grootenhuis, Nicole Blijlevens, Leontien C. M. Kremer, Eline van Dulmen-den Broeder, Hans Knoop, Jacqueline Loonen

**Affiliations:** 1 Department of Hematology, Radboud University Medical Center, Nijmegen, The Netherlands; 2 Department of Dentistry, Radboud Institute for Health Sciences, Radboud University Medical Center, Nijmegen, The Netherlands; 3 Institute for Computing and Information Sciences, Radboud University, Nijmegen, The Netherlands; 4 Department of Human Genetics, Radboud Institute for Health Sciences, Radboud University Medical Center, Nijmegen, The Netherlands; 5 Interdisciplinary Center Psychopathology and Emotion Regulation (ICPE), University of Groningen, University Medical Center Groningen, Groningen, The Netherlands; 6 Princess Máxima Center for Pediatric Oncology, Utrecht, The Netherlands; 7 Department of Pediatric Oncology/Hematology, Beatrix Children’s Hospital/University of Groningen/University Medical Center Groningen, Groningen, The Netherlands; 8 Department of Pediatric Oncology, Erasmus Medical Center, Rotterdam, The Netherlands; 9 Department of Pediatric Oncology, Erasmus Medical Center – Sophia Children’s Hospital, Rotterdam, The Netherlands; 10 Department of Medicine, section Endocrinology, Erasmus Medical Center, Rotterdam, The Netherlands; 11 Department of Internal Medicine, Leiden University Medical Center, Leiden, The Netherlands; 12 Dutch Childhood Oncology Group – Late Effects after Childhood Cancer (LATER) registry, Utrecht, The Netherlands; 13 Department of Psychology, Princess Máxima Center for Pediatric Oncology, Utrecht, The Netherlands; 14 Department Pediatric Oncology, Emma Children’s Hospital, University of Amsterdam, Amsterdam, The Netherlands; 15 Department of Pediatric Oncology/Hematology, Amsterdam University Medical Center, Amsterdam, The Netherlands; 16 Department of Medical Psychology, Amsterdam University Medical Centers, University of Amsterdam, Amsterdam Public health research institute, Amsterdam, Netherlands

**Keywords:** Methodology, Multivariable fatigue model, Associated factors, Cancer related fatigue, Chronic fatigue, Childhood Cancer survivors

## Abstract

**Background:**

A debilitating late effect for childhood cancer survivors (CCS) is cancer-related fatigue (CRF). Little is known about the prevalence and risk factors of fatigue in this population. Here we describe the methodology of the Dutch Childhood Cancer Survivor Late Effect Study on fatigue (DCCSS LATER fatigue study). The aim of the DCCSS LATER fatigue study is to examine the prevalence of and factors associated with CRF, proposing a model which discerns predisposing, triggering, maintaining and moderating factors. Triggering factors are related to the cancer diagnosis and treatment during childhood and are thought to trigger fatigue symptoms. Maintaining factors are daily life- and psychosocial factors which may perpetuate fatigue once triggered. Moderating factors might influence the way fatigue symptoms express in individuals. Predisposing factors already existed before the diagnosis, such as genetic factors, and are thought to increase the vulnerability to develop fatigue. Methodology of the participant inclusion, data collection and planned analyses of the DCCSS LATER fatigue study are presented.

**Results:**

Data of 1955 CCS and 455 siblings was collected. Analysis of the data is planned and we aim to start reporting the first results in 2022.

**Conclusion:**

The DCCSS LATER fatigue study will provide information on the epidemiology of CRF and investigate the role of a broad range of associated factors in CCS. Insight in associated factors for fatigue in survivors experiencing severe and persistent fatigue may help identify individuals at risk for developing CRF and may aid in the development of interventions.

**Supplementary Information:**

The online version contains supplementary material available at 10.1186/s12874-021-01298-7.

## Background

Childhood cancer survival rate has improved significantly over the last few decades, with currently an expected 5-year survival rate of more than 80% [1–3]. Unfortunately, survival does not come without consequences of cancer treatment. Almost three quarters of Childhood Cancer Survivors (CCS) suffers from late effects following cancer treatment which can occur years or even decades after treatment [4]. A debilitating late effect is Cancer-Related Fatigue (CRF) [5]. The National Comprehensive Cancer Network (NCCN) has defined CRF as a distressing, persistent, subjective sense of physical, emotional, and/or cognitive tiredness or exhaustion related to cancer and/or cancer treatment that is not proportional to recent activity and interferes with usual functioning [6]. It differs from fatigue experienced by healthy individuals; CRF is more severe, more distressing, leads to disability and is less likely to be relieved by rest [7]. In addition, CRF most likely has a negative impact on quality of life (QoL) but thus far this has only been investigated in subgroups of CCS [8, 9].

Previous literature did not establish consensus about the prevalence and risk factors of CRF in CCS. A systematic review investigating CRF in CCS showed a wide range in prevalence rates (0–61.7%, *n* = 18,682) [10]. In addition, a recently published guideline for the surveillance of CRF in childhood, adolescent, and young adult cancer survivors also showed a wide range of prevalence rates (10–85%, *n* = 11,628) [11]. Both studies stated that clinical and statistical heterogeneity of previous literature made it difficult to compare the results and draw conclusions regarding CRF prevalence and risk factors. To gain knowledge on the prevalence and associated factors of CRF, a sufficiently large, systematic and comprehensive multicenter collaborative project was suggested [11].

**Fig. 1:**
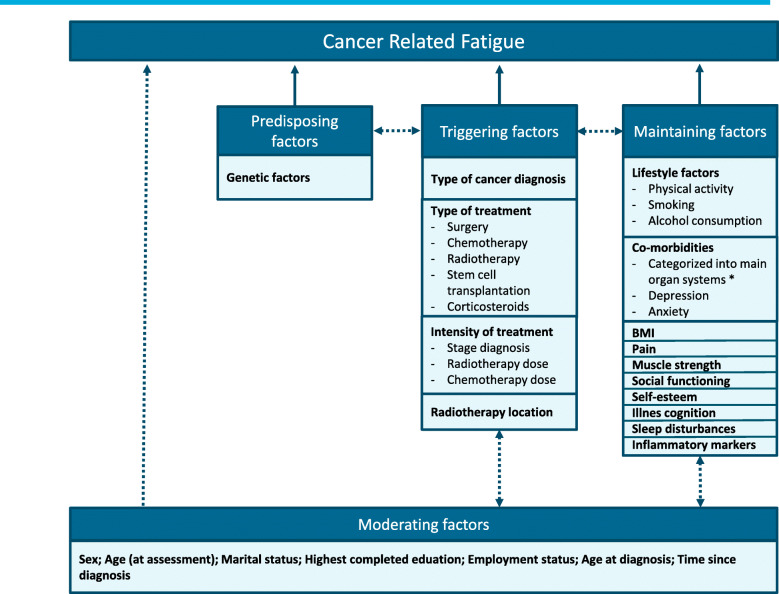
Hypothesized multivariable CRF model in CCS. Model showing associated factors of CRF divided into predisposing- (genetic factors and blood biomarkers which are thought to impact the vulnerability to fatigue), triggering- (factors related to the cancer diagnosis and treatment during childhood and are thought to trigger fatigue symptoms), maintaining- (daily life- and psychosocial factors which may perpetuate fatigue once triggered) and moderating factors (factors which might influence the way fatigue symptoms express in individuals). Continuous lines: factors that are hypothesized to be directly related to CRF. Dashed lines: factors that are hypothesized to possibly act as moderator or confounder for other factors, but might also directly be related to CRF. BMI: Body Mass Index. * Included comorbidities are categorized into the following main organ systems: Neoplasms, Cardiac-, vascular-, respiratory-, gastro-intestinal-, hepatobiliary-, renal and urinary tract-, endocrine-, musculoskeletal-, ear-, eye-, nervous system-, and other conditions

Fatigue is a subjective, multifactorial symptom. Diverse factors such as age, sex, mental status and health status have, among others, been shown to influence fatigue [12]. As these factors are closely related, it is desirable to evaluate their relationship with fatigue following cancer in a multivariable model. In this way, possible associations between factors are taken into account and confounding is corrected for. An example of such a model was presented by Bower et al. [13], including diagnosis and treatment related factors and predisposing and maintaining factors to investigate the role of neuro-immune reactions on CRF in survivors of adult-onset cancer (ACS). Another multivariable model was presented by Koornstra et al. [14] to investigate CRF in cancer patients and included a vast array of associated factors among which were comorbidities, medication use and tumor related factors. Both models emphasize the importance of a multicausal and multidisciplinary approach to investigate CRF. Studies using such models to investigate associated factors of CRF have focused on patients and survivors of adult-onset cancer [13, 14]. In the current study we will focus on CRF in CCS using a model which distinguishes between predisposing, triggering, maintaining and moderating factors (Fig. 1).

Here, we describe the methodology of the Dutch Childhood Cancer Survivor Late Effect Study on fatigue (DCCSS LATER fatigue study). The aim of the DCCSS LATER fatigue study is to examine the prevalence of and factors associated with CRF in CCS, based on the presented model. Also, the impact of CRF on QoL in CCS will be investigated. This is the first study to use a nationwide (Dutch) cohort including all tumor types to investigate the role of a broad range of associated factors of CRF in CCS. Combining these factors in one study will hopefully increase the knowledge of CRF in CCS and enable adequate identification of risk groups.

## Methods

### Study design

The DCCSS LATER fatigue study is a cross-sectional study in a nationwide cohort of Dutch CCS. It is part of the DCCSS LATER study which is a comprehensive, multidisciplinary program for patient care and research into various late effects in CCS. Where the DCCSS LATER fatigue study focuses on fatigue as a late effect in CCS, other DCCSS LATER sub-studies focus on second primary malignancies, thyroid function, hormone deficiency, metabolic syndrome, reproductive potential, bone mineral density, sexuality and psychosexual development, cardiovascular toxicity, renal effects, pulmonary dysfunction, psychosocial consequences, splenic function, hyposalivation and benign sequalae. In all pediatric oncology centers in the Netherlands data was collected from patient files, questionnaires and during a visit at the expert clinic for late effects following cancer (LATER outpatient clinic). During the visit, which took place between 2017 and 2020, participants received regular medical care and simultaneously data was collected for the DCCSS LATER study. The DCCSS LATER fatigue study was approved by the Medical Research Ethics Committee of the Amsterdam University Medical Center (registered at toetsingonline.nl, NL34983.018.10). The study was carried out in accordance with the declaration of Helsinki [15].

### Objectives

The objectives of the DCCSS LATER fatigue study are to 1) investigate the prevalence of CRF in a cohort of CCS including all cancer types and 2) determine factors which might be associated with CRF in CCS. This study will provide an estimate of overall- and treatment specific risks for CRF in CCS. This knowledge should enable identification of groups at risk for developing fatigue following cancer treatment.

### Current status of the study

At the moment of writing, data collection has already finished. Currently, the data is being cleaned by data-managers. The aim is to start with the analyses of the data in 2021.

### The study population

**Fig. 2:**
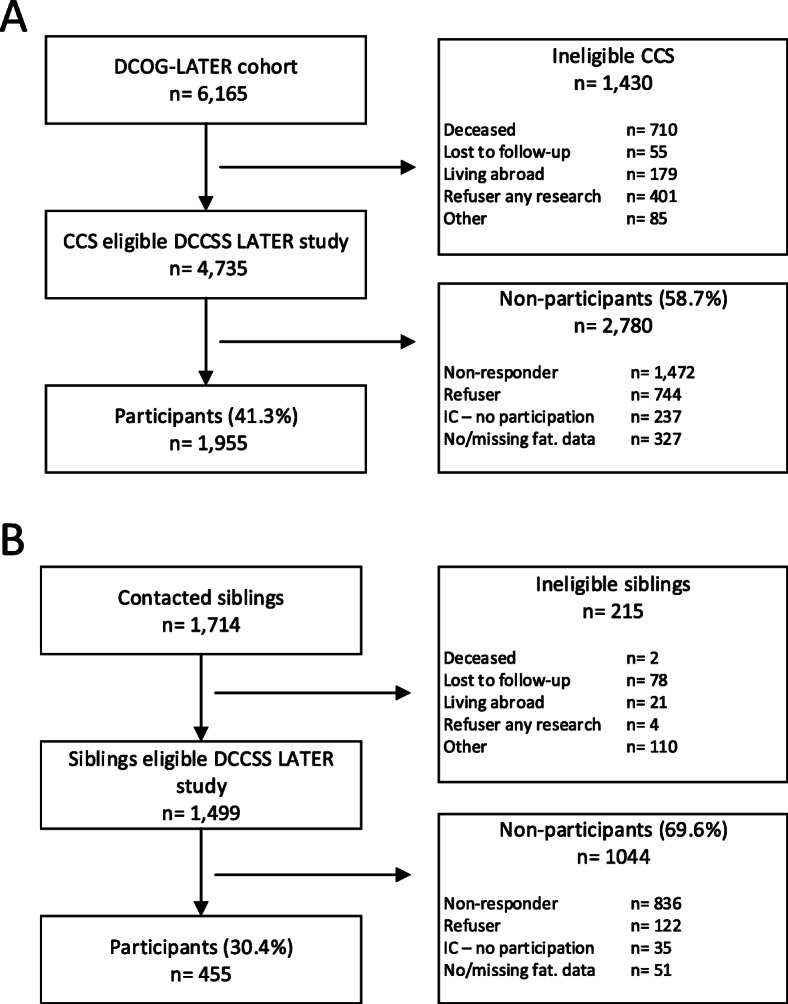
Flowcharts. **a**: Flowchart of the CCS participants. **b**: Flowchart of the sibling participants. IC – no participation: Gave consent, however did not participate. No/missing fat. Data: Did not complete the Checklist Individual Strength subscale fatigue (CIS-fatigue), or duration fatigue symptoms was unknown. When only one item of the CIS-fatigue was missing, the missing value was imputed with the mean score of the other seven items (*n* = 5 survivors and *n* = 3 siblings). Participants with two or more missing values on the CIS-fatigue were excluded

Participants of the DCCSS LATER fatigue study were included from the DCCSS LATER cohort (*n* = 6165). This is a nationwide cohort of five-year CCS diagnosed with histologically confirmed malignancies [16] or Langerhans cell histiocytosis before the age of 18 between January 1st 1963 and December 31st 2001 in the Netherlands. From this cohort, CCS living in the Netherlands who were alive on January 1, 2017, when the invitation process started, were invited to participate (Fig. 2a). Participants gave written informed consent (or their parents when aged < 16 years, *n* = 3).

#### Controls

##### Siblings

A control group consisting of siblings of CCS were included which enables matching on many unmeasured factors such as ethnicity, genetic background, culture, community, socioeconomic status and family environment. Survivors who participated in the study were asked to provide contact details of their siblings which were used to invite them to participate. Siblings, who have not had cancer and who can read and speak Dutch, and who gave written informed consent, were approached to participate in the sibling control group (Fig. 2b).

##### Population controls

Data of Dutch population controls participating in the *Lifelines project* will be used as a second control group, since siblings may be affected by the disease history of their brother or sister in some way. These participants broadly represent the general Dutch population. *Lifelines* is a multi-disciplinary prospective population-based cohort study examining in a unique three-generation design the health and health-related behaviors of approximately 167,000 persons living in the North East of The Netherlands [17]. It employs a broad range of investigative procedures in assessing the biomedical, socio-demographic, behavioral, physical and psychological factors which contribute to health and disease of the general population. When we start data analysis for the DCCSS LATER fatigue study, *Lifelines* data of approximately 90.000 participants will be made available and will then be matched on age and sex with the survivors. The Lifelines control group will be substantially larger than the CCS study group, ensuring sufficient power to analyze differences in prevalence rates. Participants with a (self-reported) history of cancer will not be included in this control group.

### Data collection

#### Fatigue

The Checklist Individual Strength (CIS) [18], a 20-item questionnaire, scored on a 7-point Likert Scale, was used to assess fatigue severity. The CIS measures several aspects of fatigue using the subscales *fatigue severity* (CIS-fatigue; 8 items)*, concentration* (5 items), *motivation* (4 items) and *physical activity level* (3 items). The total score ranges from 20 to 140 where a higher score corresponds with more problems in this area. The CIS is a reliable and valid instrument for the assessment of fatigue, with a score of 35 or higher on the *CIS-fatigue severity subscale* (range 8–56) indicating severe fatigue, which was validated in currently treated cancer patients and ACS [19].

#### Triggering factors

Factors related to the cancer diagnosis and treatment during childhood are thought to trigger fatigue. Information about the diagnosis and cancer treatment was collected by data-managers using a uniform and standardized protocol [20]. Details comprise information on treatment start and end dates, treatment type i.e. surgery, chemotherapy (CT), radiotherapy (RT) and stem cell transplantation (SCT), treatment dose of CT and RT and RT location. All treatment data cover treatments for the initial tumor and all recurrences plus RT boosts when applicable. Survivors who received radiotherapy will be categorized in groups dependent on the body part which was irradiated. We will distinguish between patients who received RT to the head, total body, spine, thorax, abdomen/pelvic region, neck, upper extremities and lower extremities (see Figure S1 in Additional file 1). Radiation-exposed volume to the head will be categorized into three groups (full-cranial-, partial-cranial- and no-cranial irradiation) following previously described methodology [21] and all irradiated regions will additionally be categorized according to dose tertiles. Survivors who received chemotherapy will be categorized in groups dependent on the specific agent they were given. We will distinguish between patients who received anthracyclines, platinum derivates, alkylating agents, vinca alkaloids and antimetabolites. Agents will additionally be categorized according to dose tertiles.

#### Moderating factors

Moderating factors might influence the way fatigue expresses in individuals. For example, females who received cancer treatment might experience different consequences compared to men who received the same cancer treatment. In that case, sex acts as a moderator leading to the development of fatigue symptoms in individuals who received cancer treatment. We believe that several demographic- and cancer treatment related factors (see Fig. 1, moderating factors) may act as a moderator for other included factors and are therefore presented as such in the hypothesized model (dashed lines from moderating factors to other factors). However, these factors may also directly influence CRF (dashed line from moderating factors directly to CRF). The exact role of these variables is yet to be determined and will be investigated by means of the planned analyses (see below). During the clinic visit, a questionnaire asking about the participant’s demographic data (see Table S1 in Additional file 1) was completed. Age at diagnosis was collected and time since diagnosis was calculated.

#### Predisposing factors

Of the possible predisposing factors of fatigue, here the role of genetic factors will be studied. Genetic factors are thought to increase the vulnerability to develop fatigue. Venous blood samples were collected from survivors (*n* = 1874) during the clinic visit after overnight fastening and stored at − 80 degrees Celsius for future evaluation. From participants of whom we were not able to collect a blood sample and from survivors after allogeneic SCT, saliva samples were collected. A genome-wide association study (GWAS) will be carried out to identify genetic variants associated with fatigue.

#### Maintaining factors

Maintaining factors are daily life- and psychosocial factors which may perpetuate fatigue once triggered. During the clinic visit, height and weight of the participants was measured to calculate their body mass index (BMI). BMI will be categorized as follows: Underweight (BMI < 18.5), normal weight (18,5 ≤ BMI < 25), overweight (25 ≤ BMI < 30), obesity (BMI ≥ 30). Grip strength was measured using an analogue hand dynamometer. Grip strength was shown to be a good reflection of a person’s muscle strength in general [22, 23]. Grip strength was measured four times (two times left arm, two times right arm) and the mean score will be used as an indication for muscle strength. Additionally, a general health questionnaire containing items about the participant’s medical history and current medical state was completed on paper prior to the clinic visit (see Table 1 in Additional file 1 for details). During the clinic visit, completeness of this questionnaire was checked by one of the research nurses and discussed with the participant when a question needed clarification. Self-reported health problems and comorbidities were validated by the physician. Comorbidities will be categorized according to previous published methodology [24] into main organ system categories: Neoplasms, Cardiac-, vascular-, respiratory-, gastro-intestinal-, hepatobiliary-, renal and urinary tract-, endocrine-, musculoskeletal-, ear-, eye-, nervous system-, and other conditions. Inflammatory markers (interleukin-1, interleukin-6, CRP) will be measured in the venous blood samples which were collected during the clinic visit. In addition, the following questionnaires were completed digitally on a laptop during the clinic visit:

##### TAAQOL

To assess QoL, the TNO (Netherlands Organisation for Applied Scientific Research) and AZL (Leiden University Medical Centre) Questionnaire for Adult’s Quality of Life (TAAQOL) was completed [25]. The TAAQOL contains twelve subscales (Gross motor function, Fine motor function, Cognitive function, Sleep, Pain, Social functioning, Daily activities, Vitality, Happiness, Aggressiveness, Depressive moods) with a total of 45 items, each scored on a 4-point Likert scale. Crude scale scores are linearly transformed to a 0–100 scale with higher scores indicating better functioning. The questionnaire has been validated in both the general population as well as in patients with chronic diseases [25, 26]. The impact of fatigue on the health related domains of QoL in CCS will be reported on in a separate study.

##### HADS

The Hospital Anxiety and Depression Scale (HADS) [27] was used to assess the level of psychological distress. It asks the participant about anxious and depressive feelings in the past 4 weeks, each containing seven items on a 4-point Likert scale. The HADS was found to be able to assess symptom severity and caseness of anxiety disorders and depression in both somatic, psychiatric and primary care patients, and in the general population [28]. A cutoff score of ≥8 for both the anxiety subscale and the depression subscale can be used to identify possible cases [28]. The HADS was validated in different age groups of the Dutch population [29].

##### RSES

The Rosenburg Self-Esteem Scale (RSES) [30] was used as a measure for self-esteem. It contains ten items, each asking the participant about global self-worth on a 4-point Likert scale. The total score ranges from 10 to 40, where a higher score corresponds with a higher self-esteem. The RSES shows satisfying psychometric properties [31].

##### PSQI

The Pittsburgh Sleep Quality Index (PSQI) [32] was used to assess sleep quality. It assesses seven components (subjective sleep quality, sleep latency, sleep duration, sleep efficiency, sleep disturbance, use of sleep medication and daytime dysfunction) which are scored 0 (no difficulty) to 3 (severe difficulty), making the total score range from 0 to 21. Psychometric properties are good and have been validated in several patient populations, including breast cancer patients [33, 34]. The PSQI can be used to screen participants for the presence of significant sleep disturbance with a *cut-off* score greater than 5 discriminating between good and poor sleepers [32].

##### Squash

The Short Questionnaire to assess health- enhancing physical activity (SQUASH) [35] was used to assess the participant’s physical activity level. It measures how frequent, how intense and how long a participant carried out a certain type of activity (physical activity from and to work, physical activity at work, physical activity during spare time and physical activity doing household activities). The SQUASH is a valid instrument for categorizing adults according to the Dutch physical activity guideline [36].

##### ICQ

The Illness Cognition Questionnaire (ICQ) was used as an instrument to assess the participant’s illness beliefs. It has 3 subscales (helplessness, acceptance and perceived benefits) each consisting of six items measured on a 4-point Likert scale, with a total score ranging from 18 to 72. Its psychometric properties were shown to be sufficient in patients with chronic diseases such as rheumatoid arthritis and multiple sclerosis [37] and in parents of a child with cancer [38].

An overview of the data we have collected and how the measures will be used to create the model parameters is shown in Table S2 in Additional file 1.

This table also shows which parameters will be available for each group of participants, i.e. CCS, sibling controls and/or Lifelines controls. Survivors who were willing to be involved in research, but who declined a visit to the LATER outpatient clinic or who were not able to come in, were invited to participate in the questionnaire-part only which could be completed digitally at home.

To examine possible selection bias in the group of survivors that agreed to participate in the DCCSS LATER fatigue study, the following, anonymized, data of the survivors who declined to participate will be retained in the central database: sex, decade of birth, childhood cancer diagnosis, decade of diagnosis, treatment with chemotherapy and /or radiotherapy (yes/no).

### Definition of fatigue

CRF, is thought to be related in time to the cancer diagnosis and treatment. However, to make the comparison with fatigue in the control groups who have not had cancer, the term CRF is not applicable. To enable a comparison between CCS and controls, we will use the term Chronic Fatigue (CF). For a reliable distinction between cases and non-cases, it is important to use a fatigue questionnaire with a validated cut-off score to indicate severe fatigue. It is also important to take into account the minimal duration of symptoms of at least 6 months which was proposed to define chronic fatigue [39] and has been used in other populations as well [8, 10, 40, 41].

We define CF as severe fatigue, indicated with a score of 35 or higher on the *CIS fatigue severity subscale* [19], which persists for at least 6 months. A question on symptom duration is part of the general health questionnaire, see Table S1 in Additional file 1.

### Statistical analyses

To examine possible selection bias between study participants and persons who declined to participate, independent t-tests will be conducted to compare the groups with respect to the variables sex, decade of birth, childhood cancer diagnosis, decade of diagnosis, treatment with chemotherapy and /or radiotherapy (yes/no).

Prevalence rates of CF within the survivors and the two control populations will be presented descriptively. Logistic regression analysis will be done with CF (yes/no) as dependent variable and group (CCS, siblings, population controls) as independent variable to determine whether prevalence rates differ between groups.

To determine factors which might be associated with CF in CCS, a multivariable logistic regression analysis will be performed with CF as dependent variable and the triggering, maintaining and moderating factors as independent variables. The predisposing genetic factors will be analyzed in a separate sub-study. Multivariable logistic regression will produce an Odds Ratio (OR) for each possible risk factor (OR with 95% CI will be presented). Each group of factors will be entered as separate block of independent variables in the analysis. Each block will be analyzed both separately as well as in combination with the other blocks to examine the association of all factors with each other. In addition, structural equation modeling (SEM) will be applied [42]. To analyze structural relationships between factors we will apply both confirmatory analyses, by assuming a particular structure between variables and testing whether this structure is supported by the available data, and more exploratory analyses that search over different structures in an attempt to detect potential causal relationships [43, 44]. The analyses will be carried out in all three study groups (with the factors available for each specific group; CCS, sibling controls and population controls) to examine if associated factors differ between groups. We will test for multicollinearity.

IBM SPSS (IBM Corp. Released 2017. IBM SPSS Statistics for Windows, Version 25.0. Armonk, NY: IBM Corp) and *R* [45] will be used for the statistical analyses.

## Results

### Study population

Characteristics of the total DCCSS LATER cohort (*n* = 6165) can be found elsewhere [46]. Of this cohort, 4735 eligible CCS participants were invited to participate in the DCCSS LATER study. Data of 1955 CCS and 455 siblings was collected. In the following months, data will be checked on correctness and cleaned. Analysis of the data is planned for 2021 and we aim to start reporting the first results in 2022.

### Expected results

We will report the prevalence of CF for CCS and two control populations. Factors associated with CF in CCS will be determined, distinguishing between predisposing, triggering, maintaining and moderating factors as presented in our model. Furthermore, we aim to create certain profiles which will help identify CCS at-risk for the development of CF.

## Discussion

We presented the methodology of the DCCSS LATER fatigue study which will investigate CF in a nationwide cohort of CCS. The study presents a model discerning predisposing, triggering, maintaining and moderating factors of CF in CCS. Investigating a broad range of possible associated factors in a single study using clearly defined methods is expected to give insight into the prevalence and associated factors of CF in CCS and will enable comparison with other studies. We hypothesize the prevalence rate of CF in CCS to be around 25%. This number is based on the combined prevalence rates of severe fatigue seen in the included studies in the previous mentioned systematic review [10] with CCS aged 16–71 years at assessment. A pilot study conducted in a partly overlapping cohort of Dutch CCS (*unpublished data, Sylvia van Deuren* et al.*)* found a similar prevalence rate of CF using the Short Fatigue Questionnaire [47, 48] to indicate CF.

In our study, the predisposing factors studied are genetic factors that might be related to how sensitive a person is to develop CF following cancer and its treatment and might also influence the persistence of fatigue. Because of the massive scope of the GWAS in which we will analyze these genetic factors, this will be done in a separate study. It is assumed that triggering factors are related to the cancer diagnosis and treatment during childhood, starting CF. Maintaining factors are daily life-, psychosocial- and inflammatory factors that may perpetuate the fatigue once triggered. Moderating factors might influence the way CF expresses in individuals. In the current study we included several demographic- and treatment related factors which might possibly act as moderators. An overview of all factors is shown in Fig. 1, wherein all variables are shown that are believed to be related to fatigue. This relation can be direct (direct lines from factors to fatigue in Fig. 1) or indirect (dashed lines between the factors in Fig. 1), for example as a confounder, moderator or mediator to other variables. Using the presented model, we aim to investigate the precise contribution of all these variables to CF in CCS. Below, we hypothesize on the potential associated factors described in the model.

### Fatigue model

The fatigue model presented is hypothetical and based on findings in the literature and on clinical experience in the LATER outpatient clinic. We hypothesize that the presented factors all play a role in the development and/or persistence of fatigue symptoms in CCS. We categorized the factors in a way we think to be appropriate and plausible. The precise role of each factor is yet to be examined. The model as presented in the current paper is meant as a starting point to create an overview of all possible associated factors. We aim, when the DCCSS LATER fatigue study is finished, to present a directed acyclic graph (DAG), a tool to help interpreting relationships in research [49], including only those variables that are directly related to CRF or act as a confounder, moderator or mediator.

### Predisposing factors

It is suggested that genetic mechanisms are involved in subjective experiences such as fatigue in cancer survivors [50]. For example, breast cancer survivors with fatigue show higher expression of genes of the pro-inflammatory system that are under control of the transcriptions factor NF-κB, compared to non-fatigued survivors [51]. Identification of genetic factors associated with CF in CCS will aid to adapt treatment based on risk models predicting susceptibility to specific late effects. Such approaches are likely to get a more prominent role in survivor care in the future [52]. In a follow up study we will investigate in detail the relation between genetics and CF in CCS. Based on the above mentioned prevalence rate, we will be able to detect Odds Ratios of 1.6 or higher per allele (allele frequency of 0.3), however with the exploratory approach of the study we believe outcomes to still be interesting. In addition, this dataset can be used for validation of previous findings in literature and can be the basis for meta-GWAS with other similar cohorts.

### Moderating factors

Several studies showed female sex to be associated with fatigue [53–56], making it likely that such an association will be present in CCS. Furthermore, age at diagnosis can influence the occurrence and severity of late effects [57, 58]. However, it is unknown whether age at diagnosis could influence the development of CF in particular. A systematic review conducted in CCS suggests age at diagnosis to not play a role in CF, however no pooled conclusion could be made [10]. Also, little is known about the relation between CF and age at assessment in CCS. It is hypothesized that a higher age at assessment is associated with CF as two studies showed higher prevalence of fatigue in older age groups of survivors compared to survivors of younger age [8, 59].

A meta-analysis in breast cancer survivors showed that having a partner decreased the risk of fatigue [60]. A questionnaire study focusing on demographic-, lifestyle- and treatment factors conducted in the DCCSS cohort showed marital status not to be related to CF (*unpublished data, Sylvia van Deuren* et al.*)*. We expect to find similar results. All these variables can directly act on the prevalence of CF or indirectly, via other variables such as type of treatment or diagnosis, in which case they act as moderator or confounder.

### Triggering factors

Type and intensity of cancer treatment have been associated with an increased risk for the development of several late adverse effects [61, 62]. A systematic review by van Deuren et al. [10] included multiple studies investigating triggering factors for fatigue in CCS and concluded that due to differences in study methodology, no conclusion could be drawn. Previous mentioned questionnaire study (*unpublished data, Sylvia van Deuren* et al.) focusing on demographic-, lifestyle- and treatment factors conducted in the DCCSS cohort showed CCS with CNS tumors to have higher odds for reporting CF compared to other childhood diagnoses. In the current study, we will investigate the association of diagnosis- and treatment-related factors with CF in CCS in more detail including all factors described in the CRF model. We will not only examine the type of treatment (surgery, RT, CT, SCT) but also the relation of treatment intensity and RT location on fatigue. Using the proposed model, we will be able to correct for multiple possible confounding factors and by doing so, we will be able to elaborate on the precise role of diagnosis- and treatment-related factors in the development of CF in CCS.

### Maintaining factors

Gielissen et al. [63] proposed maintaining factors to be responsible for the persistence of fatigue, based on a cognitive behavioral model of CRF in which it is assumed that cognitions and behavior can maintain fatigue. Factors such as, social functioning, illness cognitions and sleep disturbances are topics addressed during cognitive behavior therapy (CBT) which was shown to be an effective therapy to reduce fatigue in ACS [63]. A pilot study investigating CBT in CCS showed promising results [64] and another study showed a substantial overlap in cognitive behavioral factors that can maintain fatigue between CCS and patients with chronic fatigue syndrome or ACS [65]. This suggests that these factors also might play an important role in maintaining CF in CCS.

Physical and psychological comorbidities have also been associated with fatigue. For example, Mulrooney et al. [54] described that CCS with heart failure, pulmonary fibrosis, depression or obesity reported more fatigue and sleep disorders. Ho and colleagues [66] showed fatigue to be related to depression in survivors of both childhood- and adult onset cancer and Karimi et al. [67] investigated the relation between depression and fatigue in CCS specifically and also showed a significant association. In contrast to Goërtz et al. (*unpublished data, Yvonne MJ Goërtz* et al.), who showed that factors associated with fatigue in several chronic conditions are not disease specific, but generic. They suggest a trans-diagnostic approach for understanding fatigue in patients with chronic health conditions. Contributing to these results, Nap-van der Vlist et al. [68] suggest a trans-diagnostic approach in children with chronic conditions as well. By including several comorbidity categories in the presented multi-factorial fatigue model, we aim to determine the precise role of these health conditions in perpetuating fatigue symptoms in CCS.

Physical activity levels were shown to be associated with fatigue levels during a one-year follow-up in a mixed cohort of childhood cancer patients and survivors [69]. Muscle strength was suggested to be related to fatigue in patients with advanced cancer [70]. In the current study hand grip strength will be used as an indication for muscle strength [22]. We hypothesize physical activity and muscle strength to be negatively correlated with CF in CCS. Information about smoking and alcohol consumption was not collected from the participants in the current study and this is considered a limitation.

Previous studies suggest Inflammatory markers to play a role in the experience of fatigue symptoms in (adult) cancer patients [13, 71, 72]. However it remains unknown whether these markers might still be related to fatigue in CCS. Therefore inflammatory markers (interleuking-6, interleukin-6 and CRP) are included in our fatigue model to investigate their association with chronic fatigue in CCS.

Above, we discussed multiple factors possibly associated with CRF in CCS and included them in a model. We aimed to create a complete model but we acknowledge that there are other factors not included in the model that also might be associated with CRF and interesting to investigate. Examples are traumatic events or past history of mood disorders. Future studies might consider including these factors.

## Conclusion

Using the model as presented, the DCCSS LATER fatigue study will provide information on the epidemiology and associated factors of CF in CCS. With person-centered care getting a more prominent role in the health-care system, insight in possible risk factors for survivors experiencing CF is of great interest to identify individuals at risk for developing CF. Ultimately, this study will hopefully contribute to the improvement of current treatment protocols decreasing CF and improving quality of life in CSS.

## Supplementary Information

**Additional file 1: Figure S1.** Flowchart of the categorization process of survivors who received Radiotherapy. **Table S1.** Items in the questionnaires regarding the participant’s demographic data, medical history and current medical state. **Table S2.** Model parameters and how they were measured in the DCCSS LATER fatigue study.

## Data Availability

Data sharing is not applicable to this article as no datasets were generated or analysed during the current study.

## Abbreviations

CCS: Childhood Cancer Survivors
CRF: Cancer Related Fatigue
CF: Chronic Fatigue
NCCN: National Comprehensive Cancer Network
DCCSS LATER: Dutch Childhood Cancer Survivor Late Effect Study
QOL: Quality of life
DCOG: Dutch Childhood Oncology Group
KIKA: Stichting Kinderen Kankervrij
CIS: Checklist Individual Strength
CT: Chemotherapy
RT: Radiotherapy
SCT: Stem cell transplantation
TBI: Total body irradiation
CrRT: Cranial radiotherapy
BMI: Body mass index
GWAS: Genome-wide association study
TAAQOL: TNO (Netherlands Organisation for Applied Scientific Research) and AZL (Leiden University Medical Centre) Questionnaire for Adult’s Quality of Life
HADS: Hospital Anxiety and Depression Scale
RSES: Rosenburg Self-Esteem Scale
PSQI: Pittsburgh Sleep Quality Index
SQUASH: Short Questionnaire to assess health- enhancing physical activity
ICQ: Illness Cognition Questionnaire
OR: Odds Ratio
DAG: Directed acyclic graph
ACS: Survivors of adult-onset cancer
CBT: Cognitive behavior therapy
