# Supplementary material for: Methodology of the DCCSS later fatigue study: a model to investigate chronic fatigue in long-term survivors of childhood cancer
Source: BMC Med Res Methodol. 2021 May 16;21:106. doi: 10.1186/s12874-021-01298-7 (PMC8127233; doi:10.1186/s12874-021-01298-7)
Supplement: Supplementary file 1 — Additional file 1: Figure S1. Flowchart of the categorization process of survivors who received Radiotherapy. Table S1. Items in the questionnaires regarding the participant’s demographic data, medical history and current medical state. Table S2. Model parameters and how they were measured in the DCCSS LATER fatigue study. [file 12874_2021_1298_MOESM1_ESM.docx]

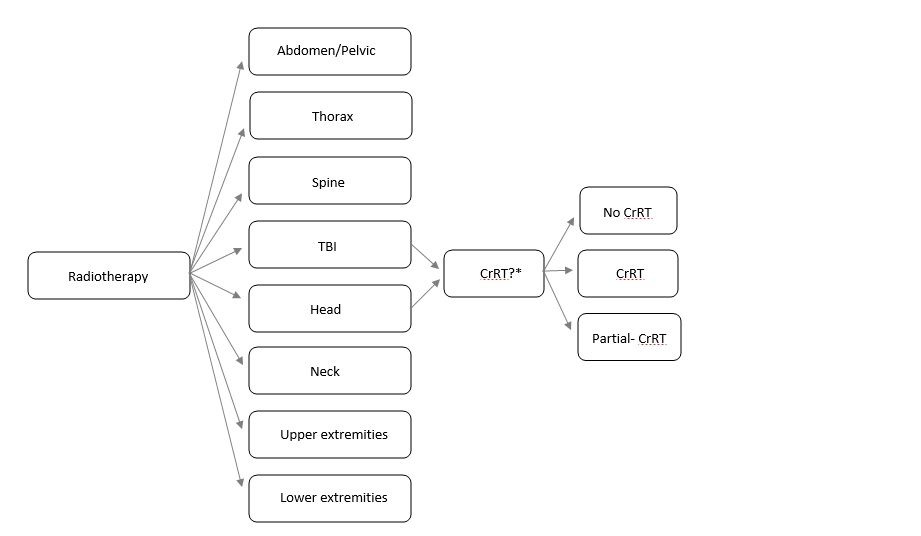
**Additional file 1. Additional figures and tables**

**Survivors who received radiotherapy directed to the head – including those who received total body irradiation (TBI) – will be assigned to one of three subgroups: full-cranial volume (full-cranial radiotherapy (CrRT); defined as 100% of the cranium in field), partial-cranial volume (partial-CrRT; defined as any CrRT with less than 100% of the cranium in field), and radiotherapy to the head without cranial involvement (no brain tissue in the field; not considered CrRT).*

**Figure 1. Flowchart of the categorization process of survivors who received Radiotherapy**

**Table 1. Items in the questionnaires regarding the participant’s demographic data, medical history and current medical state.**

| **Subject** | **Item in the general health questionnaire** |
| --- | --- |
| **General Health** | |
| Medical status | Could you please indicate if you currently or ever did suffer any of the the conditions stated below. If so, could you please state in what year you got the diagnosis and if you are currently using medication for it (and if so, which medication).  The following conditions were listed: heart attack, angina pectoris, heart valve defect, pericarditis, cardiomyopathy, heart failure, cardiac arrhythmia, heart defect since birth ….*, other heart disease…*, stroke, vascular abnormality ….*, condition with increased risk for thrombosis (protein C deficiency, protein S deficiency, factor V Leiden mutation, other ….*), hypertension, high cholesterol, stomach or intestine problems, lung disease ….*, kidney problems (for example kidney stones, too much protein in your urine, cysts) ….*, adrenal glands problems ….*, liver problems ….*, musculoskeletal problems (for example arm/leg/elbow/knee) ….*, diabetes, epilepsy, cataract, tinnitus, reduced height growth, hypothyroidism, hyperthyroidism, thyroid nodule, other thyroid conditions ….*, other condition related to hormone regulation ….*, other condition ….* |
| Medical status | Do you, more than 3 times a year, experience problems with your respiratory system? |
| Medical status | Have you ever had an infection of the urinary tract (including a fever)? If so, how many times? 1 time, 2-5 times or more than 5 times? |
| Medical status | Do you use hearing aid? |
| Fatigue status | Do you experience fatigue problems and if so, for how long do these fatigue problems exist (number of weeks/months/years)? |
| Medication use | Do you use medication, other than for a condition you may experience as described above, more than once a week (for example aspirin, ibuprofen)? If so, please list them here. |
| **Demographic data** | |
| Age | What is your date of birth? |
| Age | At what date did you fill in this questionnaire? |
| Sex | Are you a male or a female? |
| Education status | What is your highest completed level of education? (answer options vary from primary school to university) |
| Employment status | Are you currently employed? If so, what work do you do? |
| Marital status | Are you currently in a relationship? |

*This table shows the items of the general health questionnaire in detail. *Participants could, if applicable, write the name of a condition at the dashed line.*

**Table 2. Model parameters and how they were measured in the DCCSS LATER fatigue study.**

| **Parameter** | **Measure** | **Available for** |
| --- | --- | --- |
| **Fatigue** | | |
| Fatigue severity | CIS | CCS, Sibling controls, Lifelines controls |
| Fatigue duration | General health questionnaire | CCS, Sibling controls, Lifelines controls |
|  |  |  |
| **Predisposing factors** | | |
| Genetic factors | Blood sample | CCS (subgroup) |
|  |  |  |
| **Triggering factors** | | |
| Cancer diagnosis | Patient record | CCS |
| Cancer treatment | Patient record | CCS |
|  |  |  |
| **Maintaining factors** | | |
| Physical activity ^c^ | SQUASH | CCS, Lifelines controls |
| Somatic comorbidities | General health questionnaire | CCS, Sibling controls ^b^ |
| Depression ^c^ | HADS | CCS, Sibling controls ^b^ |
| Anxiety ^c^ | HADS | CCS, Sibling controls ^b^ |
| BMI | Height and weight measured during visit ^a^ | CCS, Sibling controls, Lifelines controls |
| Muscle strength ^c^ | Hand dynamometer | CCS |
| Pain ^c^ | TAAQOL, subscale pain | CCS, Sibling controls ^b^ |
| Self-esteem ^c^ | RSES | CCS, Sibling controls ^b^ |
| Social functioning ^c^ | TAAQOL, subscale social functioning | CCS, Sibling controls |
| Sleep disturbances ^c^ | PSQI | CCS, Sibling controls ^b^ |
| Illness cognition ^c^ | ICQ | CCS, Sibling controls |
| Pro-inflammatory markers ^c, d^ | Blood sample | CCS |
| **Moderating factors** | | |
| Sex (male/female) | Patient record / General health questionnaire | CCS, Sibling controls, Lifelines controls |
| Age at assessment | Patient record / General health questionnaire | CCS, Sibling controls, Lifelines controls |
| Age at diagnosis | Patient record | CCS |
| Time since diagnosis | Patient record / General health questionnaire | CCS |
| Marital status | General health questionnaire | CCS, Lifelines controls |
| Education status | General health questionnaire | CCS, Lifelines controls |
| Employment status | General health questionnaire | CCS, Lifelines controls |

*CCS: Long term survivors of childhood cancer, CIS: Checklist Individual Strength, SQUASH: Short Questionnaire to assess health- enhancing physical activity, HADS: Hospital Anxiety and Depression Scale, TAAQOL: TNO and AZL Questionnaire for Adult’s Quality of Life, RSES: Rosenberg Self-Esteem Scale, PSQI: Pittsburgh Sleep Quality Index, ICQ: Illness Cognition Questionnaire. ^a^ For CCS participants who will not visit the clinic, height and weight will be asked in a questionnaire. For the Lifelines participants, height and weight is measured during a visit at the Lifelines clinic. ^b^ Parameters were measured in the Lifelines controls as well, however using a different questionnaire. ^c^ Parameters will be used as a predictor for CF in the DCCSS LATER fatigue study, however will be described as primary outcome in other DCCSS LATER substudies. ^d^ Interleukin-1, Interleukin-6, C-reactive protein (CRP)*
